# Triple Akt inhibition as a new therapeutic strategy in T-cell acute lymphoblastic leukemia

**DOI:** 10.18632/oncotarget.3260

**Published:** 2015-03-02

**Authors:** Alice Cani, Carolina Simioni, Alberto M. Martelli, Giorgio Zauli, Giovanna Tabellini, Simona Ultimo, James A. McCubrey, Silvano Capitani, Luca M. Neri

**Affiliations:** ^1^ Department of Morphology, Surgery and Experimental Medicine, University of Ferrara, Ferrara, Italy; ^2^ Department of Biomedical and Neuromotor Sciences, University of Bologna, Bologna, Italy; ^3^ Institute for Maternal and Child Health, IRCCS “Burlo Garofolo”, Trieste, Italy; ^4^ Department of Molecular and Translational Medicine, University of Brescia, Brescia, Italy; ^5^ Department of Microbiology & Immunology, Brody School of Medicine, East Carolina University, Greenville, NC, USA; ^6^ LTTA Center, University of Ferrara, Ferrara, Italy

**Keywords:** T-acute lymphoblastic leukemia, Akt, Perifosine, GSK690693, MK-2206

## Abstract

T-cell acute lymphoblastic leukemia (T-ALL) is an aggressive neoplastic disorder in which chemotherapy resistance and refractory relapses occur, with a poorer prognostic outcome.

Constitutively active PI3K/Akt/mTOR pathway is a common feature of T-ALL upregulating cell proliferation, survival and drug resistance. This pathway is currently under clinical trials with small molecules inhibitors (SMI).

To verify whether a multi-inhibition treatment against Akt protein could enhance the efficacy of individual drug administration and overcome drug resistance as well as to obtain a decrease in single drug concentration, we tested on T-ALL cell lines the effects of combined treatments with three Akt inhibitors with different mode of action, GSK690693, MK-2206 and Perifosine.

In cells with hyperactivated Akt, combined administration of the drugs displayed a significant synergistic and cytotoxic effect and affected PI3K/Akt/mTOR pathway at much lower concentration than single drug use. Highest synergistic effect for full inhibition of Akt was also related to the timing of every drug administration. Furthermore the triple treatment had greater efficacy in inducing cell cycle arrest in G_0_/G_1_ phase and both apoptosis and autophagy.

Targeting Akt as a key protein of PI3K/Akt/mTOR pathway with multiple drugs might represent a new and promising pharmacological strategy for treatment of T-ALL patients.

## INTRODUCTION

T-ALL is an aggressive and heterogeneous blood malignancy disease deriving from T-cell progenitors in the thymus, with multiple, prognostically relevant genetic aberrations [1] and is characterized by the over-production of immature white blood cells [2].

T-ALL accounts for 10–15% of pediatric and 25% of adult ALL cases [3]. With the current intensified multi-agent chemotherapy protocols, the 5-year event-free survival (EFS) of children with T-ALL has reached 70–75%, whereas the EFS is 30–40% for adults below 60 years of age, and 10% above this age [4]. However, these therapies are highly toxic and moreover, relapsed patients often develop resistance to chemotherapy and have an extremely poor prognosis [5]. Heterogeneity within T-cell leukemia can cause avoidance of inhibited nodes and selection of subpopulations of inhibitor-resistant cancer cells [6].

Constitutively active PI3K/Akt/mTOR signaling is observed in many types of solid and blood tumors, including T-cell acute lymphoblastic leukemia (T-ALL), where it portends a poorer prognosis and negatively influences response to therapeutic treatments [7].

The phosphoinositide 3-kinase (PI3K)/Akt/mammalian target of rapamycin (mTOR) signal transduction cascade controls a wide range of physiological cell processes, including proliferation, survival, differentiation, metabolism, autophagy, angiogenesis, exocytosis, and motility [8]. Activation of PI3K recruits cellular protein kinases that in turn activate downstream kinases, including the serine/threonine kinase Akt. Phosphorylation of Akt activates several substrates, including the mTOR complex 1 (mTORC1) and induces subsequent phosphorylation of downstream targets such as S6K. The activation of mTORC1 results in increased translation and protein synthesis [9]. A second complex comprising mTOR, known as mTORC2, more recently described, appears to act as a feedback loop via Akt phosphorylation on Ser 473 [10].

The PI3K/Akt/mTOR network is involved in T-ALL survival and drug-resistance and could be targeted by small molecules inhibitors (SMI) [11]. The PI3K/Akt/mTOR inhibitors are currently being developed for clinical use either as single agents or in combination with conventional chemotherapy for T-ALL patient treatment [12].

There is a growing interest in multi-component chemotherapy: the combined delivery of multiple drugs is an attempt to overcome drug resistances and to improve clinical outcome.

Therefore we sought to determine whether the combination of drugs with the same target of action, i.e. Akt, will result in a more significant biological effect, as an antiproliferative therapy in order to overcome the risk of cell growth escape phenomena.

To this aim we employed three drugs directed against Akt but with a totally different mode of action. GSK690693 is a small molecule ATP-competitive inhibitor of the pro-survival kinase Akt, is a pan-Akt kinase inhibitor, has been preclinically tested in osteosarcoma and ALL xenografts and is now in phase I of clinical trials in sarcomas, neuroblastoma, non-glioblastoma brain tumors and lymphoma [13, 14].

MK-2206 is a novel, orally active, potent and selective allosteric Akt inhibitor, that inhibits the activities of all three human Akt (recombinant full length) isoforms, Akt1, 2, and 3 with 50% inhibitory concentration (IC_50_) values of 8, 12, and 65 nM, respectively and which is in phase II of clinical trials and has been tested on acute myelogenous leukemia [15] and on solid tumors, including malignant glioma, melanoma and squamous cell carcinoma [16, 17].

Perifosine, is an orally available alkylphospholipid, Akt inhibitor, that blocks the recruitment of the pleckstrin homology (PH) domain of Akt kinase, to prevent its membrane localization and subsequent activation [18, 19]. It is currently in phase III clinical development for treatment of colorectal cancer (CRC, in combination with capecitabine) and of multiple myeloma (MM, in combination with bortezomib and dexamethasone) [20], of chronic lymphocytic leukemia [21] and relapsed or refractory acute leukemia (AML or ALL) [22].

Here, we documented that multiple inhibition of Akt protein was cytotoxic against T-ALL cells over-expressing Ser 473 p-Akt and had synergistic effects, with more potent efficacy than single or double compound administration at the same concentration. Treatment of T-ALL cells with the combination of three drugs caused a potent cell cycle arrest in G0/G1 phase, apoptosis and autophagy. Moreover, 6 h of Perifosine pre-treatment followed by the combined administration of MK-2206 and GSK690693, was necessary for the complete switch off of the activated protein.

Our findings suggested that multiple Akt targeting by drugs with different mechanism of action could represent a new promising treatment for T-cell acute lymphoblastic leukemia patients with PI3K/Akt/mTOR pathway hyperactivation.

## RESULTS

### PI3K/Akt/mTOR pathway activation status in T-ALL cell lines

By Western blot analysis, we first evaluated the phosphorylation status of key proteins of the PI3K/Akt/mTOR pathway in a panel of T-ALL cell lines (JURKAT, MOLT-4, CEM-S, CEM-R, PEER and BE-13). PEER and BE-13 cells are characterized by the NUP214-ABL1 fusion gene mutation, that is the most frequent and highly specific ABL1 fusion protein for T-lineage acute lymphoblastic leukemia, that transforms T-cells and is a constitutively active tyrosine kinase with oncogenic potential [23].

**Figure 1:**
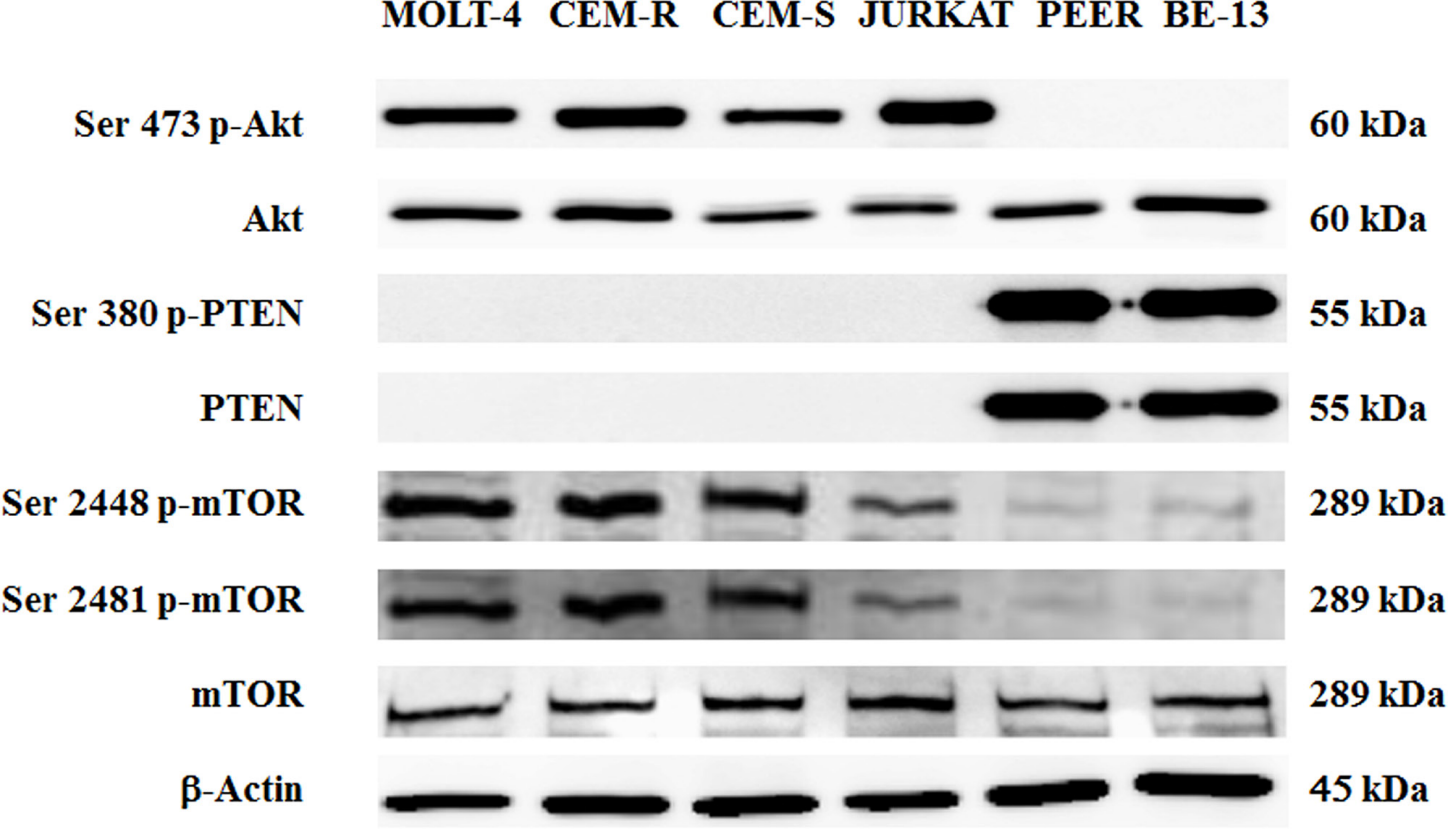
Expression and phosphorylation status of Akt, PTEN and mTOR in T- ALL cell lines Western blot analysis of T-ALL cell lines to detect the expression and phosphorylation levels of Akt, PTEN and mTOR. Twenty-five μg of protein was blotted to each lane. Antibody to β-actin served as a loading control.

Whereas PEER and BE-13 cells did not displayed phosphorylated Akt, the other four cell lines displayed an hyperphosphorylated form, which is indicative of constitutive activation of PI3K signaling pathway (Fig. 1).

Moreover, we confirmed as previously reported [24, 25] the absence of PTEN protein expression in most T-ALL cell lines analyzed, except for PEER and BE-13.

Accordingly to the above observations, Ser 2448 and Ser 2481 p-mTOR (readout of mTORC1 and mTORC2 activity) were hyperphosphorylated in NUP214-ABL1 negative cells.

In PEER and BE-13 cell lines, PTEN was phosphorylated at Ser 380, a marker of PTEN post-translational inactivation and consequent PI3K pathway activation [26]. However, despite this observation neither Akt nor mTOR appeared hyperphosphorylated.

### Multiple Akt targeting had higher cytotoxic effect and synergized only in Ser 473 p-Akt expressing cells

**Figure 2:**
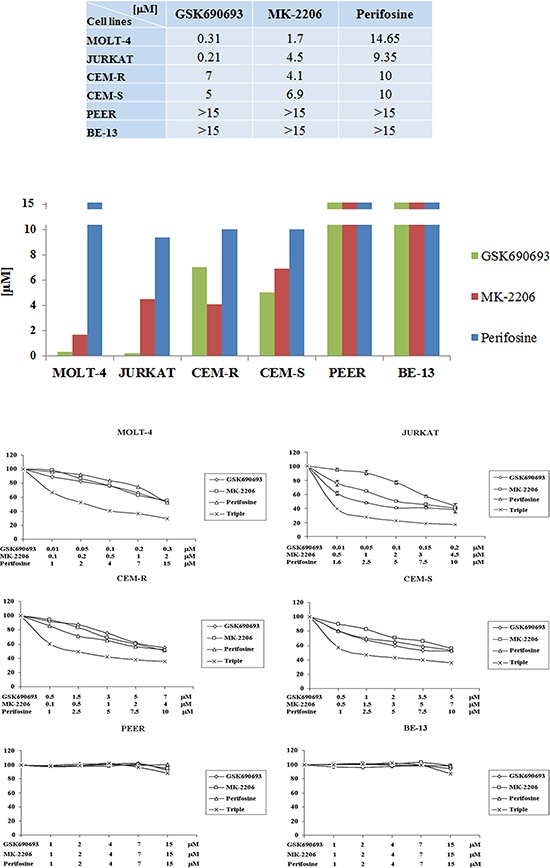
Cytotoxicity of Perifosine, MK-2206 and GSK690693 in T-ALL cell lines **A** MOLT-4, JURKAT, CEM-R, CEM-S, PEER and BE-13 cell lines were treated for 24 h with increasing concentrations of the drugs, ranging from 0.1 to 20 μM, to determine the IC_50_ value. Results are the mean of three different experiments. SD was less than 10%. **B** MTT assay of T-ALL cell lines treated with Perifosine, MK-2206 and GSK690693, either alone and in triple combination for 24 h. Concentration of each drug is reported under the graph. One representative experiment of three is shown.

We examined by MTT assay the IC_50_ values of each drug on the six T-ALL cell lines. After 24 h of treatment, cell lines displayed different sensitivity to the single drugs. GSK690693 ranged from 0.31 or 0.21 μM, in MOLT-4 and JURKAT, to 7 or 5 μM in CEM-R and -S, respectively. MK-2206 IC_50_ ranged from 1.7 to 6.9 μM. Perifosine required higher concentration to obtain IC_50_ and ranged between 9.35 and 14.65 μM (Fig. 2A) and this phenomenon is well known and has already been described [27, 28]. In both PEER and BE-13 the IC_50_ values of all the drugs was higher than 15 μM (Fig. 2A).

We next studied if the simultaneous administration of GSK690693, MK-2206 and Perifosine could lead to a similar cytotoxic effect on the T-ALL cell lines with a significant decrease of the concentration of every single drug due to the synergy of the three compound combination.

Therefore we treated cells with drugs administered together for 24 h, using the IC_50_ value as the highest one and decreasing progressively up to 1/20 of the IC_50_ value. MTT assays were then performed.

As shown in Fig. 2B, all the four more responsive cell lines showed the synergistic cytotoxicity of the triple drug combination, very significant in MOLT-4 and JURKAT cells. We calculated the cell index value (CI) with Calcusyn Software, to quantify the combined effects of the drugs, such as synergism or interference. We did not maintain a constant ratio since it was necessary to fine tune each drug concentration to better understand the synergistic or interfering effect and to avoid a too high cytotoxicity depending on a single drug administered at a fixed constant ratio. The data analysis (not shown) indicated strong synergisms in all cell lines, even more evident in MOLT-4 cells, with the best CI value of 0.101 corresponding to the combination of 0.05 μM GSK690693, 0.2 μM MK-2206 and 2 μM Perifosine.

### Triple Akt hit increases the inhibition of the PI3K/Akt/mTOR signaling pathway

To verify if the multiple and simultaneous *in vitro* treatment with MK-2206, GSK690693 and Perifosine could lead to a modulation of PI3K/Akt/mTOR pathway, we checked the phosphorylation status of key components of this signaling cascade in our panel of more responsive cell lines. In particular we analyzed p-Akt, its downstream target, GSK3 α/β, and the ribosomal protein S6 kinase, readout of mTORC1 activity, after 30 min of drugs exposure.

GSK690693 and Perifosine were used at 1/2 of the IC_50_ concentration, whereas MK-2206 was used at 1/5 of IC_50_, since half of MK-2206 IC_50_ concentration was enough to completely abolish the Ser 473 Akt phosphorylation already at 30 minutes.

**Figure 3:**
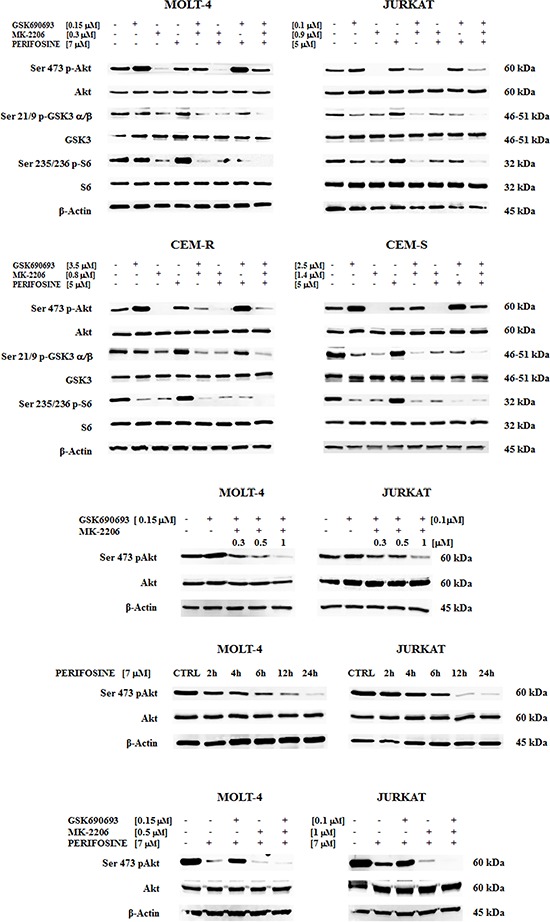
Multiple Akt inhibition affects PI3K/Akt/mTOR pathway and the Akt inhibition is time-dependent **A** Western blot analysis of Akt drug sensitive T-ALL cell lines for total and phosphorylated form of Akt and of its downstream substrate GSK3 α/β and of mTOR downstream target S6. Samples were treated for 30 minutes with GSK690693, MK-2206 and Perifosine, alone or in double or triple combinations. **B** Akt protein inhibition as detected by its phosphorylation status. MOLT-4 and JURKAT cells were treated for 30 minutes with a combination of a fixed concentration of GSK690693 and three different concentrations of MK-2206. **C** Akt phosphorylation levels in cells treated with 7 μM Perifosine at different time points. **D** p-Akt status in MOLT-4 and JURKAT cells pre-treated for 6 h with 7 μM Perifosine followed by GSK690693 and MK-2206 administered for 30 minutes. Twenty-five μg of protein was blotted to each lane. β-actin served as a loading control. For all panel one representative experiment of three is shown as well as cell lines are representative also of the others if not shown.

Akt phosphorylation was affected in different ways by single drug administration: in all cell lines MK-2206 very significantly reduced p-Akt, Perifosine only slightly reduced it and GSK690693 on the contrary increased the protein phosphorylation. The latter one is an already described phenomenon [29]. This increase of Akt phosphorylation diminished the observable effect of double or triple compound combination, since p-Akt was not significantly reduced, unless when using MK-2206 in double exposure (Fig. 3A).

On the contrary, even after such a short time of treatment, in all of the four cell lines it was very evident the efficacy of the multiple hit on Akt. The triple administration of the drugs completely abolished the phosphorylation on the downstream targets, Ser 21/9 p-GSK3 α/β and Ser 235/236 p-S6, with a much superior efficacy of the triple exposure when compared with the single or with the different double combinations (Fig. 3A). The total amount of the proteins was unchanged in all the treatments (Fig. 3A).

### Pre-treatment with Perifosine enhance synergistic effect

Given that the GSK690693 drug alone led to Ser 473 p-Akt increase, whereas MK-2206 alone almost turn off the signal, we sought to explore if we can find a compound combination capable of synergistically dephosphorylate Akt.

We first tested if there is any concentration capable to modulate Akt phosphorylation in JURKAT and MOLT-4 cells. Therefore GSK690693 was administered at 1/2 of the IC_50_ value (0.1 μM for JURKAT and 0.15 μM for MOLT-4 cells) and MK-2206 was contemporary given at increasing concentrations (0.3–0.5–1 μM). After 30 minutes of exposure, Western blot was performed. The best drug combination to observe p-Akt modulation resulted to be 1 μM MK-2206 for JURKAT and 0.5 μM for MOLT-4 cells (Fig. 3B).

We then analyzed by Western blot the phosphorylation levels of Akt after treatment with 7 μM Perifosine at different time points. In both cell lines the drug affected in a time-dependent manner the Ser 473 Akt phosphorylation (Fig. 3C).

Finally, we merged the two previous assays pre-treating cells for 6 h with Perifosine before a 30 min administration of GSK690693 and MK-2206.

As shown in (Fig. 3D), in 6 h Perifosine pre-treated cells, the administration of GSK690693 reduced Ser 473 p-Akt hyperphosphorylation. The combination of all three drugs allowed to obtain a full Akt dephosphorylation in both MOLT-4 and JURKAT cells, thus showing that full Akt inhibition with low drug doses is not only concentration but also time and drug sequence dependent.

### The triple Akt inhibition induces cell cycle arrest and causes autophagy and pro-apoptotic effects in T-ALL cells

The significant *in vitro* antitumor activity of the triple anti Akt SMI drug combination on T-ALL cells led us to investigate the mechanisms of its antileukemic efficacy. To assess the effects of the combined treatment on the PI3K pathway, we analyzed the effect of triple treatment on cell cycle progression, given the importance of the PI3K/Akt/mTOR signaling pathway in the regulation of cell proliferation [12].

**Figure 4:**
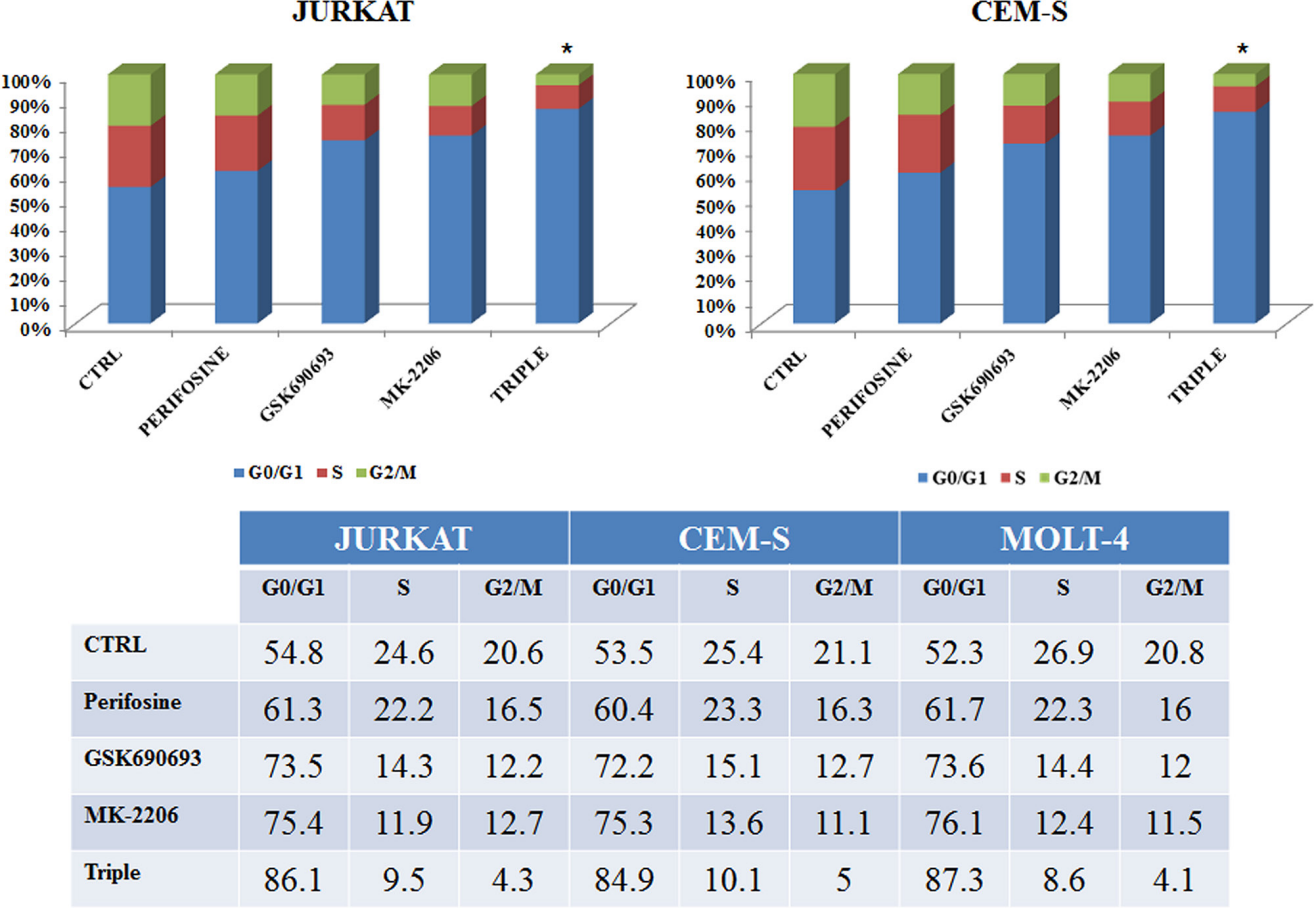
Multiple Akt inhibition induces cell cycle arrest Flow cytometric analysis of PI-stained samples was performed on JURKAT and CEM-S cells after 24 h of treatment with Perifosine, GSK690693 and MK-2206 administered both alone or in triple combination, always used at the IC_50_ value. In the table are reported the values of every cell cycle phase obtained in all treatments, with the addition of MOLT-4 cells. Asterisks indicate statistically significant differences with respect to untreated cells (*p < 0.05). One representative experiment of three is shown as well as cell lines are representative also of the others not shown.

Flow cytometric analysis of PI-stained samples in JURKAT and CEM-S cells was performed. Cells were treated with single and triple administration of drugs for 24 h. The multiple anti Akt treatment increased the percentage of cells in G0/G1 phase of cell cycle, with a parallel decrease of both S and G2/M phases (Fig. 4).

It has been reported that Akt targeted drugs induce autophagy in human glioma and T-ALL cells [30, 31].

**Figure 5:**
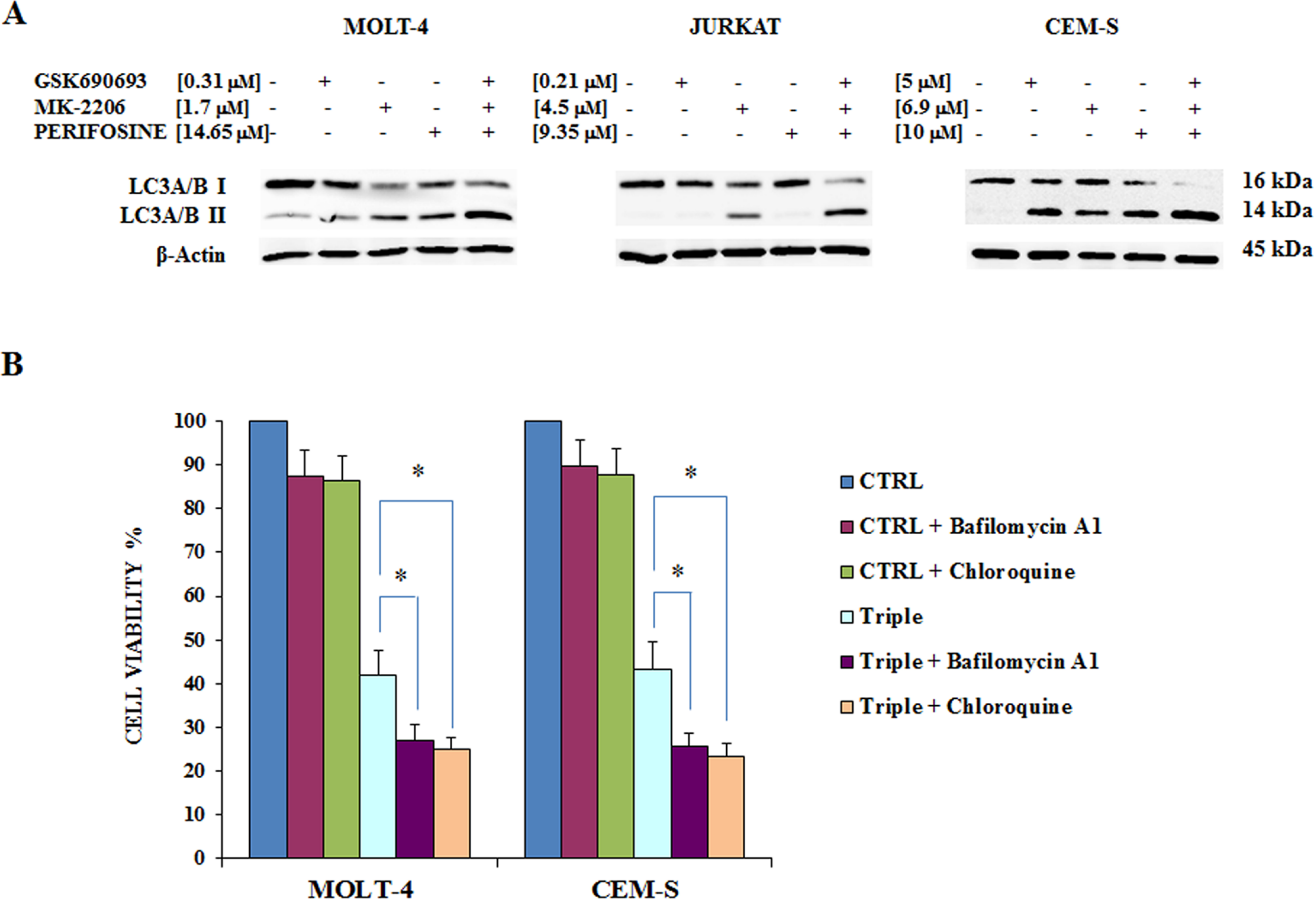
Triple Akt inhibition induces enhanced autophagy **A** The effect of the three drugs administered alone or in combination on autophagy, after 24 h of treatment, in MOLT-4, JURKAT and CEM-S cell lines, documented by the lipidation of the autophagy marker LC3A/B. The increase in the triple treatment is well evident. Antibody to β-actin served as a loading control. **B** MTT assays documenting the effects of Bafilomycin A1 and Chloroquine on viability of MOLT-4 and CEM-S cells treated for 24 h with Perifosine, GSK690693 and MK-2206 administered in combination. Results are mean of three different experiments ± SD. Asterisks indicate significant differences (*p < 0.05). One representative experiment of three is shown as well as cell lines are representative also of the others not shown.

Therefore, we investigated whether the triple treatment enhanced autophagy in our T-ALL cell panel. The exposure of cells to 24 h of the combined treatment increased the amount of lipidated (14-kDa form) LC3A/B isoform, a well-established autophagy marker, detected by western blot in MOLT-4, JURKAT and CEM-S cells (Fig. 5A).

Interestingly it has been previously reported that inhibiting autophagy with Chloroquine sensitizes non small cell lung cancer cells (NSCLC) to combined treatment with Akt inhibitors [32].

We then inhibited autophagy using Bafilomycin A1 or Chloroquine and measured cell viability after 24 h of treatment by MTT assay. Bafilomycin A1 or Chloroquine, when used alone, did not significantly affect cell viability. However when each drug was combined with triple Akt inhibition, it was possible to detect a significantly increased cytotoxicity in CEM-S and MOLT-4 cells (Fig. 5B). These findings suggest that autophagy could protect T-ALL cells by the cytotoxic effects of the Akt inhibitors.

**Figure 6:**
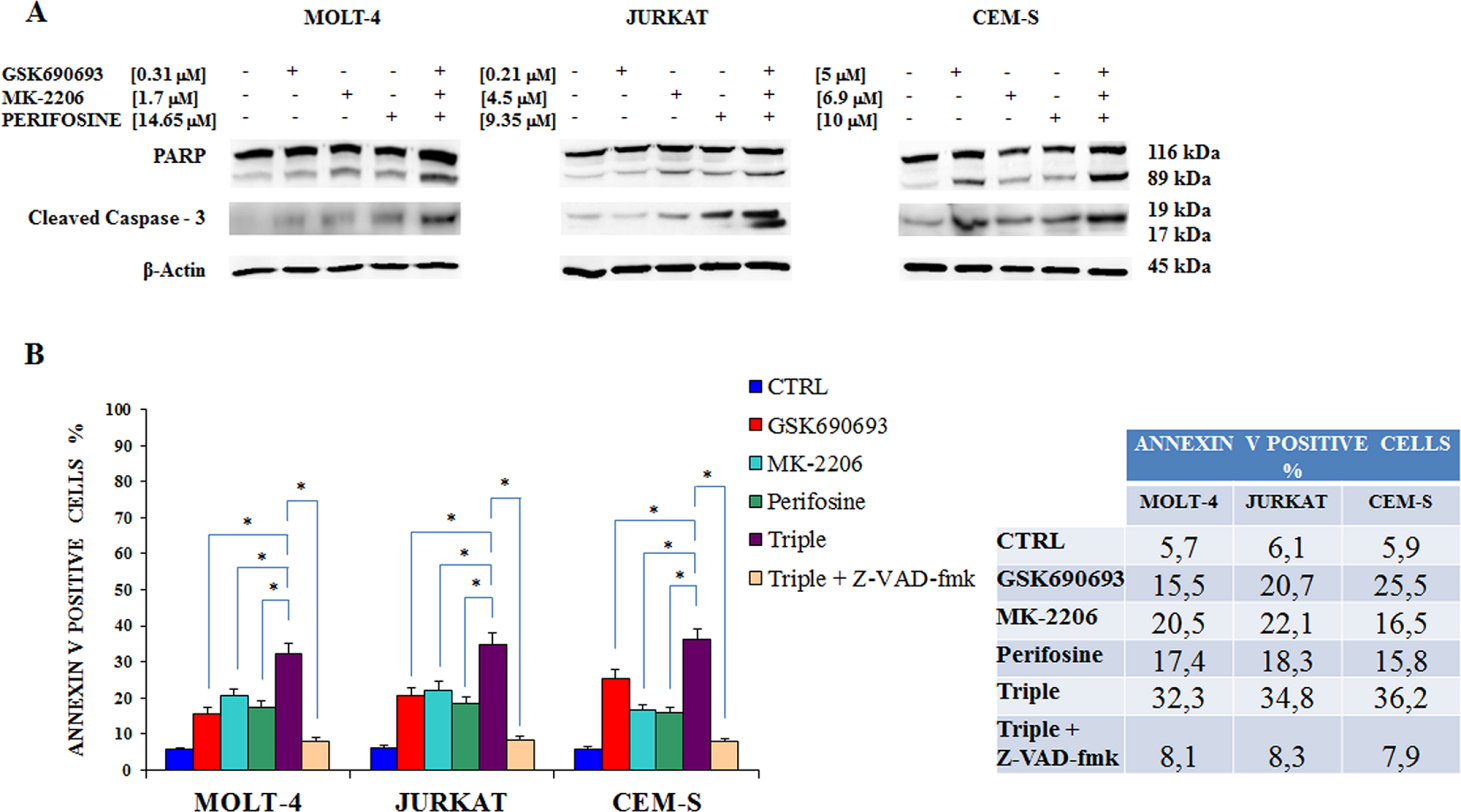
Combined Akt inhibition increases apoptosis **A** Western blot analysis documenting the increase of PARP or of caspase-3 cleavage in MOLT-4, JURKAT and CEM-S cell lines, after 24 h of multiple drugs administration when compared with single exposure. Antibody to β-actin served as a loading control. **B** Flow cytometric analysis of MOLT-4, JURKAT and CEM-S cell lines treated with Perifosine, GSK690693 and MK-2206 administered alone and in combination for 24 h is reported. Triple drug exposure was also performed in the presence of 50 μM Z-VAD-fmk, a pan-caspase inhibitor. In the table are reported the percentages of annexin V positive cells after each treatment. Samples were incubated with Annexin V-fluorescein isothiocyanate. Results are the mean of three different experiments ± SD. Asterisks indicate statistically significant differences (*p < 0.05). One representative experiment of three is shown as well as cell lines are representative also of the others not shown.

We next verified the induction of cleaved PARP, a well established apoptotic marker [33], in JURKAT, MOLT-4 and CEM-S cell lines by western blotting after 24 h of treatment. The apoptotic effect of the single drug administration was visible in each cell lines compared to the control, but was even more evident in MOLT-4 treated with MK-2206 and CEM-S treated with GSK690693. Interestingly, the triple administration of the drugs displayed a higher cleaved PARP in all the three cell lines (Fig. 6A). To further strengthen this observation we also analyzed caspase-3, which plays an essential role during apoptotic cell death [34]: also with this protein the triple exposure to drugs induced an increased caspase-3 cleavage (Fig. 6A). The percentages of apoptotic cells was examined by flow cytometry after staining with annexin-V-FITC and PI. Flow cytometric analysis confirmed that multiple treatment induced a greater, statistically significant, increase in apoptosis compared to single drugs (Fig. 6B). In addition we studied if Z-VAD-fmk, a pan caspase inhibitor [35] would affect the apoptotic process, as detected by annexin V staining: cell death was sensitive to the administration of 50 μM Z-VAD-fmk, that inhibited apoptosis in triple drug exposed cells.

### The multiple treatment has the capability to inhibit ERK pathway

We finally then focused our attention on the MEK/ERK signaling pathway, which plays an important role in cell proliferation and growth [36] and potentially mediates resistance to drug-induced growth inhibition [37].

For this reason, we examined the p-ERK 1/2 phosphorylation status in JURKAT, MOLT-4 and CEM-S cells, after 24 h of treatment with the three drugs.

**Figure 7:**
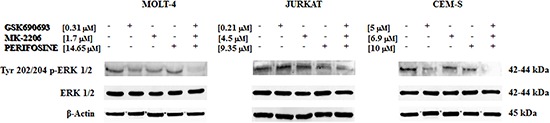
Multiple anti Akt drug treatment affects also MEK/ERK pathway Western blot analysis for Tyr 202/204 p-ERK 1/2 phosphorylation status in MOLT-4, JURKAT and CEM-S cells, after 24 h of treatment with the three drugs alone and in combination. The triple treatment almost abolished p-ERK staining. Twenty-five μg of protein was blotted to each lane. β-actin served as a loading control. One representative experiment of three is shown as well as cell lines are representative also of the others not shown.

Interestingly, the multiple treatment was capable to downregulate the phosphorylation state of Tyr 202/204 p-ERK 1/2 with a superior efficacy than single drugs, whereas the amount of total protein remains unchanged (Fig. 7).

## DISCUSSION

PI3K/Akt/mTOR signaling upregulation is very common in T-ALL, being detectable in 70–85% of the patients [38], and portends a poorer prognosis [39]. Although the survival of younger patients with acute leukemia has improved in the early 21st century, mutations event can occur at any stage of the disease and negatively influences the response to therapeutic treatments because lead to resistance to therapy.

A major challenge remains the lifelong morbidity suffered by patients treated with current chemotherapy regimens. For long-term survivors, acute and lasting toxicities remain important issues underlining the critical need of more effective and selective personalized and targeted therapies, and treatment strategies.

A large variety of inhibitors have been widely used both *in vitro* and *in vivo* in preclinical settings of acute leukemias, where they blocked cell proliferation and induced, sometimes, apoptosis and/or autophagy [40–43]. Several studies have highlighted that both PI3K and mTOR modulators could synergize with a wide range of drugs that are currently in use for treating acute leukemias, including chemotherapeutic drugs [44, 45]. Drugs dual targeting PI3K/Akt/mTOR pathway at various points of the signaling cascade are under evaluation in preclinical models and clinical trials, but the observation that not even the combined inhibition of Akt and mTOR is enough to completely turn off the pathway during chronic treatment is very intriguing [46].

Despite several chemotherapy combinations were tested *in vitro* and are in clinical trials for the treatment of acute lymphoblastic leukemia, a new and promising innovative idea, for the individualization of the therapies, could be represented by hitting the same target with multiple specific drugs with different mechanisms of action.

In this study, we demonstrated the efficacy of multi-inhibition of the same target, i.e. the Akt protein as a pivotal molecule of the PI3K/Akt/mTOR signaling pathway with three drugs with a totally different mechanism of action.

The triple administration of GSK690693, MK-2206 and Perifosine in cells with Ser 473 p-Akt hyperphosphorylation was cytotoxic and synergic at lower doses when compared with the IC_50_ values. We showed the relevance of a fine tuning of the single drug concentration to obtain the best synergistic effect. Single inhibition of Akt was lower when compared with every dual inhibition, which in turn was lower than the triple one.

We also demonstrated the importance of two issues: the compound concentration and the timing of drugs administration. About the first one, two of the drugs, MK-2206 and GSK690693 were very efficient acting synergistically even at low doses, whereas Perifosine administered together did not add its efficacy in the 10–20 μM range. The second issue disclosed that MK-2206 and GSK690693 may act very rapidly (minutes), but Perifosine requires a longer period of time (hours), to enter in action and to really synergize with MK-2206 and GSK690693.

These findings indicate that Akt inhibition mechanism may be compatible with a fine tuning of the concentrations of the single drugs, that may help with low doses to reduce the side effect of the therapy, and with crucial time points in which administer the drugs, that otherwise may improve the efficacy of the therapy. These needs are present in several reports already cited in the introduction [15–17, 20–22]. Further studies can sort out this issue.

We also documented the increasing of cleaved PARP and caspase-3, well known markers of apoptosis, thus showing this as a mechanism for the cytotoxicity of anti Akt drugs.

Both autophagy and apoptosis are well-known biological processes for programmed cell death, that play essential roles in development, tissue homeostasis and disease, with interactions among components of the two pathways [47]. Autophagy is a cellular catabolic degradation process that results in the autophagosomic-lysosomal degradation of cytosolic proteins and other cellular components [48].

Interestingly, here we demonstrated the increment of autophagy in multiple anti Akt drugs treated samples. Moreover, either the Bafilomycin A1 or the Chloroquine autophagy inhibitors, sensitized cells to treatment. These findings demonstrated that autophagy is a prosurvival mechanism that protected cells from Akt inhibitors induced cytotoxicity and the use of autophagy inhibitors may be considered in future drug combinations, also in clinical trials.

Compensatory upregulation of parallel signaling through the MEK/ERK1/2 pathway in response to PI3K/Akt inhibition, is an emerging theme in cancer cell signal transduction, because it potentially mediates resistance to drug-induced growth inhibition [37]. Indeed, several recent reports have highlighted the importance of functional crosstalks between the MEK/ERK1/2 and PI3K/Akt signaling networks, in response to individual pathway inhibitors [37, 49–53].

The MEK/ERK1/2 pathway often reflects the rebound of Akt inhibition, developing or amplifying hyperactivation. Unexpectedly, our results displayed an inhibition of ERK activation in correspondence of multiple drugs administration. It is tempting to speculate that the multi-Akt inhibition could represent an effective treatment to block crosstalk between PI3K/Akt/mTOR and Raf/MEK/ERK reducing tumor growth and cells proliferation.

Genomic DNA mutational analysis of Akt1 exons and adjacent splice sites evidenced a somatic point mutation in the Pleckstrin Homology domain (PH) of Akt1 gene: a lysine substitution for glutamic acid at the amino acid 17 (E17K). This genetic alteration was originally identified in solid tumors [54, 55]. Cystallography studies demonstrated that E17K mutation modified Akt1-PH conformation and disrupted ionic interaction with Lys 14 in the lipid-binding pocket of Akt1 [56]. This mutation have effects on sensitivity to targeted pathway inhibitors, since it has been recently reported that the Akt1- E17K mutant cells maintained higher levels of Akt1 Ser 473 phosphorylation even with increasing concentrations of MK-2206 [57].

Very recently a total of five Akt1 variants such as E17K, E17S, E319G, L357P, and P388T were found to exert deleterious effects on the protein structure and function. Furthermore, the molecular docking study indicated the lesser binding affinity of inhibitor with the mutant structure than the native type. Moreover, the findings strongly indicated that screening for Akt1, E17K, E17S, E319G, L357P, and P388T variants may be useful for disease molecular diagnosis and also to design the potential Akt inhibitors [58].

Therefore it appears of great importance to hit Akt as a pivotal molecule in cancer development and progression with drugs acting with multiple mode of action, to overcome the potential resistance due to mutations in the site of action of the single drug.

There is great clinical interest in determining whether mutations in the PI3K signaling pathway can serve as biomarkers to predict sensitivity to drugs targeting the pathway. Thus, the presence of Akt mutations or of PI3K/Akt/mTOR hyperactivation should be taken in account when establishing a therapeutic regimen for the patient. This is an important issue toward the personalized medicine, since it is useless to activate a treatment if the correspondent pivotal cytotoxic target is absent [59].

In conclusion, our preclinical finding strongly demonstrated that a multiple inhibition of Akt could represent a new promising therapeutic strategy to overcome relapse or resistance in the treatment of T- ALL patients with hyperactivated PI3K/Akt/mTOR signaling pathway.

## MATERIALS AND METHODS

### Materials

RPMI-1640 medium, fetal bovin serum (FBS), penicillin and streptomycin were from Lonza (Lonza Milano SRL, Milan, Italy). Perifosine, GSK690693, and MK-2206 were from Selleck Chemicals (Houston, TX, USA). For cell viability determination, Cell Proliferation Kit I (MTT) was purchased from Roche Applied Science (Basel, Switzerland). Annexin V/7-AAD detection kit was from Merck-Millipore (Darmstadt, Germany). Akt-1, Ser 473 p-Akt-1, Tyr 202/204 p-ERK 1/2 and ERK 1/2 primary antibodies were from Santa Cruz Biotechnology (Santa Cruz, CA, USA) while all the other antibodies were from Cell Signaling Technology (Danvers, MA, USA), including the rabbit secondary antibody. The mouse secondary antibody, Bafilomycin A1, Chloroquine and Z-VAD-fmk were from Sigma Aldrich (Milan, Italy). Signals were detected with the ECL Plus reagent purchased from Perkin Elmer (Boston, MA, USA).

### Cell culture and Western blot analysis

The T-ALL cell lines were obtained from Deutsche Sammlung von Mikroorganismen und Zellkulturen GmbH (Braunschweig, Germany). JURKAT, MOLT-4, CEM-S (drug sensitive) and CEM-R (CEMVBL100, drug-resistant cells overexpressing 170-kDa P-glycoprotein) were grown in RPMI 1640 medium supplemented with 10% heat-inactivated fetal bovine serum (FBS); PEER and BE-13 were grown in RPMI 1640 with 20% FBS. All the media were supplemented with 100 units/ml penicillin and 100 mg/ml streptomycin. The cells were grown at a density of 0.5 to 2 × 10^6^ cells/ml and were incubated at 37°C with 5% CO_2_. Western Blot analysis was performed by standard methods as described elsewhere [60].

### Cell viability analysis

MTT (3-(4,5-dimethylthythiazol-2-yl)-2,5-diphenyltetrazolium bromide) assays were performed as previously described [61].

### Cell cycle analysis

Cell cycle analysis was performed using propidium iodide (PI)/RNase A staining by flow cytometry according to standard techniques, as described elsewhere [62]. In brief, after 24 h of drug treatment, cells were harvested, centrifuged at 300 × g for 5 min and washed once with 1X PBS. After fixing them with 70% ethanol at 20°C, cells were centrifuged at 300 × g for 5 min and washed once with 1X PBS. Then 100 μl of propidium iodide (PI)/RNase A staining was added to each tube with an incubation of 30 min at room temperature in the dark. Samples were then analyzed according to the manufacturer's instructions.

### PI/Annexin V assay

To determine the extent of apoptosis induction, flow cytometric analysis of Annexin V-FITC/PI-stained samples was performed [20]. Samples were analyzed on an FC500 flow cytometer (Beckman Coulter) with the appropriate software (CXP, Beckman Coulter) [60].

### Combined drug effect analysis

The combination effect and the potential synergy of Perifosine, MK-2206 and GSK690693 were evaluated from quantitative analysis of dose–effect relationship, as described previously [63].

For each combination experiment, a combination index (CI) number was calculated using the BiosoftCalcuSyn software (Biosoft, Cambridge, UK). This method of analysis generally defines CI values from 0.9 to 1.1 as additive, from 0.3 to 0.9 as synergistic and below 0.3 as strongly synergistic, whereas values over 1.1 are considered as antagonistic.

### Statistical evaluation

The data are presented as mean values from three separate experiments ± SD. Data were statistically analyzed by a Dunnet test after one-way analysis of variance (ANOVA) at a level of significance of *p* < 0.05 vs control samples [64].
